# Towards equitable and trustworthy genomics research

**DOI:** 10.1016/j.ebiom.2022.103879

**Published:** 2022-02-12

**Authors:** Jerome Atutornu, Richard Milne, Alesia Costa, Christine Patch, Anna Middleton

**Affiliations:** a Engagement and Society, Wellcome Connecting Science, Wellcome Genome Campus, Cambridge CB10 1SA, UK; b School of Health and Sports Sciences, University of Suffolk, Ipswich, IP4 1QJ; c Kavli Centre

**Keywords:** Diversity, Inclusion, Equity, Justice, Genomics research

## Abstract

The representation of traditionally scientifically underserved groups in genomic research continues to be low despite concerns about equity and social justice and the scientific and clinical need. Among the factors that account for this are a lack of trust in the research community and limited diversity in this community. The success of the multiple initiatives that aim to improve representation relies on the willingness of underrepresented populations to make data and samples available for research and clinical use. In this narrative review, we propose that this requires building trust, and set out four approaches to demonstrating trustworthiness, including increasing diversity in the research workforce, and meaningful engagement with underrepresented communities in a culturally and linguistically appropriate manner. Capacity building globally will ensure that actual and perceived exploitation and ‘helicopter’ research could be eliminated.

## Introduction

Advances in genomic sequencing technologies have enabled improvements in disease diagnosis, treatment and personalised care.^1^ Despite these improvements, a lack of diversity persists in the research that informs genomic medicine. There are clear ethical justifications for increasing diversity in order to ensure just distributions of the benefits of genomic medicine.^2^ In addition, the scientific success of genomic projects depends on the availability of large amounts of data that can be shared across scientific and geographic boundaries.^3^

Various international initiatives are geared towards addressing imbalances in data.^1^^,^^3^, ^4^, ^5^ Efforts are also underway to connect these endeavours to ensure that these data can be accessed across institutional and national boundaries. For example, the European Beyond One Million European Genomes Alliance links the electronic health records and genomic sequence results of at least one million people across Europe.^6^ On the African continent, the African Genome Variation Project has catalogued the genomic profile of 100 individuals each from 10 ethnic groups for 2.5 million genetic variants.^7^ Additionally, deep whole-genome sequencing of African individuals who exhibited complex region-specific structures has also been done.^8^ The largest initiatives in Africa are currently funded by the NIH (USA) and Wellcome (UK), through the H3Africa consortium.^9^ Within the H3Africa consortium, there is work on developing and harmonising standards for ethics and data sharing across African countries.^10^^,^^11^ Further expansion of efforts to establish a resource of 3 million African genomes has recently been proposed.^12^

In what follows, our aim is to characterise the problem of lack of diversity in genomics, discuss possible reasons for it and proffer some practical steps towards addressing it. We conclude that the lack of engagement of traditionally scientifically underrepresented groups with genomics could be addressed by culturally and linguistically appropriate engagement practices. Our focus is primarily on the inclusion of diversity that is representative of the African continent. While many of the findings and conclusions from this work may also be pertinent to other groups traditionally under-represented in scientific research, it is important to recognise the critical role of specific cultural, historical and geographical contexts in shaping engagement with genomics.^13^^,^^14^

## Search strategy and selection criteria

Data for this narrative review were identified by searches of MEDLINE, PubMed, Web of Science and references from relevant articles using the search terms “diversity”, “inclusion”, “equity”, “justice”, “genomics research”. Only articles published in English relevant to equity in human genomics research were explored. Except for seminal and historic sources, published articles that were included spanned 2016 to 2021. We recognise that our search strategy and selection criteria will inevitably miss literature that has been published in other databases. We also acknowledge that by choosing databases that mostly consider genomics within the context of Western, Anglophone approaches to medicine, science and philosophy we have inadvertently excluded valuable literature that will be framed differently.

## Lack of diversity in genomic databases

Much genetic variation is rare and specific to different genetic ancestry groups.^15^ Large amounts of data are therefore needed so that credible inferences can be made regarding the link between a genetic variant and a disease of interest, since making inferences from studies of only one genetic ancestry group could be inaccurate.^3^

It is currently estimated that data from people of European ancestry accounts for at least 78% of genome-wide association studies while individuals of African ancestry account for 2.4%.^16^ Over 70% of samples included in genome-wide association studies (GWAS) come from only three countries – the USA, Iceland and the United Kingdom.^16^ In the latter, groups who are traditionally scientifically underrepresented continue to be so in datasets such as UK Biobank.^17^ Hence, while people of African ancestry (comprising of black African, black Caribbean and black ‘other’) constitute 3.4% of the UK population (according to the 2011 census), their representation in UK Biobank constitutes only 1.6%. Conversely, white British participants constitute 94% while making up 91.3% of the population.^18^

The lack of ancestral diversity in genomic datasets may mean that studies based on them may be less able to yield results that are meaningfully transferable for all members of these populations as a whole.^19^^,^^20^ In addition to this, there is no universally accepted, consistently applied method for categorising genomic data, particularly by ethnicity and race, thus there is much room for confusion by what is even meant by diversity. This is problematic, both from a clinical and scientific perspective, as we elaborate below, but more fundamentally in terms of the ability of genomics research to achieve the core ethical values of equity and justice. These principles are enshrined in the global ethical and bioethical frameworks developed over the past century. Article 27 of the United Nations’ Universal Declaration on Human Rights (UDHR), for example, states that: “Everyone has the right to participate in the cultural life of the community, to enjoy arts and to share in scientific advancement and its benefits”.^21^ The influential US Belmont Report, which outlines ethical principles to guide biomedical and behavioural research similarly emphasises the importance of justice and the question of “who ought to receive the benefits of research and bear its burdens”.^22^ Until recently however, questions of justice and genomics have often received comparatively little attention.^23^ As a matter of justice, it is fair to expect that the advantages that accrue from scientific discoveries should benefit those most in need and not just the scientifically well represented. Diversity in genomics can contribute to developing just and equitable genomic medicine and efficiently allocating scarce healthcare resources.

## The scientific and clinical case for equity

In the African continent alone, the extent of genetic diversity is enormous.^8^ There is now a recognition of a subdivided population structure as a result of historic human migration patterns, with Africa comprising at least 11 ancestral groups, compared to 12 ancestral groups identified in the rest of the world.^7^^,^^8^ This diversity often leads to differences in the variants associated with specific disorders and genes, making it more challenging to find the link between genetic variants and disease in these populations.^7^ It implies that causal links between genetic variants and disease cannot be trusted in medicine if the data that guides variant interpretation does not include populations from diverse ancestral backgrounds. Put bluntly, it means that a patient may be given the wrong genetic diagnosis or risk profile of disease. Increasing diversity in genomic research can thus give greater insight into the implications of human history and ancestral influences on health and disease.^24^ Specifically, it can contribute to understanding differences in the global distribution of disease, better prescribing, and drug development, reducing misdiagnosis and more accurate prediction of disease risk.

There are well-established differences in disease prevalence between human genetic ancestry groups. For example, Tay-Sachs disease is more common in people of Ashkenazi Jewish descent,^25^ cystic fibrosis is more common in people of Northern European ancestry^26^ and beta thalassemia is more common in people of Greek, Italian, Middle Eastern, Asian and African descent.^27^ The finding that variants in the apolipoprotein L1 (APOL1) gene are associated with an increased risk of kidney disease^28^ can help to explain why kidney disease is more prevalent among people of African ancestry. While this variant is often absent in individuals of non-African descent, it is common among individuals of African descent. The high frequency of this variant is believed to be because it confers protection against trypanosomiasis or African sleeping sickness.^29^ This knowledge offers the opportunity for a better understanding of kidney disease^30^ but may also be useful in the allocation of medical resources to these populations.

Improved understandings of human biology through genomic studies of diverse populations may lead to better prescribing and drug development.^31^^,^^32^ For example, a variation in the PCSK9 gene is associated with a reduction in low-density lipoprotein cholesterol concentration and a reduction in the risk of coronary heart disease.^33^ This variant is found in higher frequencies in individuals with African ancestry than in people of European descent.^34^ Two drugs resulting from this discovery have been approved (evolucumab and alirocumab) for the reduction of low-density lipoprotein cholesterol concentration.^35^ Another drug, inclisiran, has also shown very good results in this context.^36^ Thus, this exemplifies the fact that different ethnic populations may have different genetic variations that can affect health and the treatment of disease and provides further support for the need for diversity in genomic research, so that effective therapies can be researched for all, instead of the select few for whom access is enabled.

A further advantage of genomic studies of diverse populations may be the prevention of misdiagnosis. One of the criteria used in identifying a variant that causes a disease is its rarity. Therefore, it is easy to mistakenly identify a variant as disease-causing if the database used for such inferences does not contain sufficient information from ethnically diverse populations.^37^ Historically, this has led to the misdiagnosis of some patients of African ancestry. For example, variants in people of African origin were classified as disease-causing for hypertrophic cardiomyopathy using samples that were mostly European.^38^ These variants, which are rare among Europeans, were subsequently found to be higher in frequency among African Americans and prevalent among people of African ancestry and were therefore unlikely to be disease-causing.^38^

Finally, the availability of diverse genomic data may contribute to improved applicability of genomic risk prediction. While for most monogenic/Mendelian diseases, a genetic variant regarded as pathogenic causes disease across populations, the picture is somewhat different when it comes to complex diseases. Polygenic risk scores (PRS) are increasingly used to calculate the risk of complex diseases in individuals.^39^ However, these computations may not be transferable across genetic ancestry groups. The prediction accuracy of PRS for several anthropometric and blood panel traits has been found to be as much as 4.5-fold lower in people of African ancestry than in Europeans.^40^ A 10-fold overestimation of schizophrenia risk has been found among Africans and African-Americans compared to Europeans.^41^ The inclusion of diverse populations is thus central to the interpretive accuracy of these tools and their translation into clinical practice.^42^

In this section we have highlighted some examples of how a lack of diversity in genomic data creates problems for equitable delivery of genomic medicine. In the next section we acknowledge that in order to be able to increase diversity in genomic data, public audiences have to be willing to donate their data – and this is directly connected to issues of trust.

## Trust and inequality in data

The development of more diverse genomic research in order to deliver fair and equitable genomic medicine, however, relies on the willingness of diverse public groups to donate data and biosamples. The success of genomics will thus depend on both its ability to deliver improved healthcare across population groups and its acceptance by publics. A recent systematic review into the readiness of the British National Health Service (NHS) to deliver its Genomic Medicine Service concluded that in addition to demonstrating clinical utility and cost-effectiveness, patient involvement and engagement would be key to successful implementation of the service.^43^

All human genomic data become available initially through the donation of samples by individuals in various contexts, whether as patients, customers or research participants having genetic testing, participating in biobank research or donating blood.^44^ One important factor shaping the availability of data is thus the willingness of members of the public to donate. This willingness is shaped by multiple factors – including people's familiarity or experience with genetics and their perception of the power of genetic information.^45^ However, in terms of increasing the participation of traditionally scientifically underrepresented groups in genomic medicine and research, it is critical to address the question of trust.

## Trust

Globally, trust is a key factor shaping the willingness to donate, yet levels of trust in those involved in genomic data collection and use vary substantially. Across 22 countries in the Your DNA, Your Say study of public attitudes towards genomic data sharing (*n* = 37,000 nationally representative public audiences from 22 countries), less than half the overall sample reported trusting someone other than their own doctor with their DNA and health information, with levels of trust highest in China, India, the United Kingdom and Pakistan, and lowest in Egypt, Russia, Germany and Poland.^46^ Within countries as well, levels of trust vary – in the UK, Australia, Canada and the USA, Your DNA, Your Say findings suggest that those most likely to trust are male, under 50, and likely to have some familiarity of genetics.^46^

As with genomics research more generally however, evidence of public perceptions of trust in genomics is heavily skewed towards a few countries, notably Europe, Australia, and the USA. In these countries, research suggests overall support for genomic data initiatives, but that a lack of trust is associated with a reluctance to participate.^47^ A systematic review of studies of African Americans’ beliefs and attitudes about genomic studies showed an overall theme of a lack of trust for research and medical communities, even though there was a recognition of the critical need for participating in such studies.^48^ One key factor shaping this lack of trust is the legacy of historical ethical failings in research affecting non-white populations in the USA. Prominent among these are the Tuskegee syphilis experiments, the Henrietta Lacks case and, more recently, the improper use in genetic research of biosamples of the Havasupai tribe.^49^^,^^50^ Studies among American participants suggest that these cases have understandably left a legacy of suspicion and mistrust both of government policies and the motives of the scientific world, and that some African Americans do express concerns that the Tuskegee abuses could happen to them, while others raise fears about who benefits from genomic studies, similar to the Henrietta Lacks case.^51^ In addition, however, genomics research also intersects with existing racial and ethnic inequalities and forms of exclusion. Thus, attitudes towards research are shaped by worries about financial profit being made at the expense of African Americans,^51^ concerns about privacy, and fear that health and genetic data may be used to discriminate against them within and outside health settings.^52^ Thus, while studies such as the AllofUs precision medicine initiative have invested substantial effort in reaching underrepresented communities, they still have some distance to travel in terms of assuaging concerns.^14^

On the African continent, fewer studies are documented for us to review in the databases we searched; in the studies we viewed concerns exist around the collection of potentially sensitive samples such as blood, the quantities taken, the use to which they are put and their export across territories.^53^ Past unethical scientific behaviour on the continent, may again engender mistrust: for example, after 15 years of litigation the pharmaceutical company Pfizer recently paid out compensation to the families of Nigerian children who allegedly died from being administered experimental drugs without proper consent.^54^ In addition, there are substantial historic reasons for potential mistrust in scientific endeavours, particularly those associated with former colonial powers. Historically, colonisation (and colonial science) has always been viewed as extractive of physical and human resources, most heinously in the case of the slave trade,^55^ and as reifying particular notions of ethnic or racial identity and characteristics.^56^ Examples abound, but the forced sterilisation of Herero women of Namibia in the early 1900s by German colonial administrators in furtherance of racist ‘scientific’ efforts to prevent mixed-race marriages is a particularly egregious example of why suspicions may persist.^57^

Fears therefore exist that these unequal colonial relationships will be re-established.^58^ These concerns are exacerbated by so-called ‘helicopter science’^59^ and concerns about national ownership of samples, the lack of recognition of local scientists and exploitation.^60^ Genomics research on the continent has often involved the large-scale export of samples to institutions in richer countries, often with no benefit to local institutions, researchers, participants and health priorities.^61^ In this context, as in the US setting,^14^ the concept of genetic and data sovereignty has been discussed and contested. Genetic sovereignty holds that the patterns of genetic variations in populations (such as Africans) have not only scientific value but also commercial and symbolic value, and that these need to be protected against exploitative ‘prospectors’.^58^

## Building trust, being trustworthy

While trust is an important factor in supporting diverse participation in genomic research, it is only part of the story. Specifically, measures of ‘trust’ focus on potential participants. In doing so, they construct a problem of a ‘trust deficit’ that needs to be remedied. For both ethical and practical reasons, this is problematic. Low levels of trust may be entirely appropriate among populations with a long history of valid reasons not to trust. In such a situation, it is incumbent upon those leading genomics research to demonstrate that trust in them is well-placed and unlikely to be betrayed. This has the additional advantage of bringing the question of trust within the remit of researchers themselves, as the question becomes not how *to build* trust, but how *to demonstrate* that trust in research is merited - to demonstrate trustworthiness. In this section, we elaborate proposals for ways in which researchers may demonstrate trustworthiness.

Unlike trust, trustworthiness has - until lately - received comparatively little attention in relation to genomics. This recent work suggests the importance of transparency about the social value of research and who is involved in the use of data, as well as highlighting the importance of ethical protections – for example the ability to withdraw data or opt-out.^62^ Establishing trustworthiness then, means being in a position to demonstrate concordance with these values – showing how research will benefit society as a whole, being clear about who should and should not be accessing data and working with communities to ensure that their values and ethical concerns are understood and reflected in the structures of research.

Four approaches to this can be identified as potentially important, related to the structure and conduct of scientific activity, and the relationship between researchers and researched populations.

As discussed above, initiatives to increase the global representativeness of genomic data are underway. However, it is also essential that those who donate data can see that this data is used for their benefit and that of others. Structurally, this means addressing the potential impact of the *preferred cohort* effect.^3^ Due to the history of genomics and the established data infrastructure, researchers may, pragmatically, be more likely to focus on data from established well-characterised datasets to satisfy reviewers and allow studies to be evaluated in terms of previous findings. Within the context of GWAS, there is scientific efficiency to undertaking studies on a more homogeneous sample. Consequently, as data from diverse populations become increasingly available, action is needed to ensure it is used, and to avoid it being evaluated as ‘supplementary’ to a European-ancestry ‘standard’. This can have the unintended consequence of additional scrutiny for novel findings that may emanate from non-European cohorts.^3^

In addition to ensuring the use of data, it is also important to consider and be transparent about *who* is using data. From an equity perspective, this means engaging with wider concerns about the lack of diversity at all levels of research, including not only participants, but also researchers and even review-panels for research proposals and data access committees for data sharing.^63^^,^^64^ Failure to do this deprives the scientific community of different perspectives and expertise, while perpetuating perceptions of ‘helicopter’ research. Thus, the equitable delivery of genomic research and medicine services would also depend on the structural changes to reflect ethnic diversity. Such changes might include ensuring diverse research teams, not as a matter of indulgence or tick box exercise, but one that recognises that diverse groups perform better than homogenous groups.^65^

Also of note is the need to pay attention directly to the relationship between researchers and communities and the reality of establishing the terms of the trust relationship. This includes consideration and acknowledgement of perceived power dynamics and (often unequal) privilege together with a recognition that these need to be articulated, debated and discussed with humility and respect. It is increasingly recognised that the views of potential participants should be sought during the design of research, particularly where this research seeks to engage with historically under- represented populations. Such community engagement is vital in demonstrating trustworthiness because it helps to ensure that research is concordant with the values and interests of the community, and further contributes to reducing the impression of ‘helicopter research’.^66^^,^^67^ Such detailed, initiative specific consultations should ideally be supported by larger scale work with the general population to establish the relationship between research agendas and people's values and priorities. To date, while the attitudes of publics towards genomics and genomic data sharing have been researched across parts of the world, this has mostly concentrated on higher income countries, with little published work with publics in or originating from sub-Saharan Africa (that is searchable within scientific databases available in this review).

Finally, consultation and engagement are also important in tackling a second, less commonly acknowledged challenge – that of language. In UK clinical genetics services, patients might reasonably indicate that their preferred language for consultation is not English.^68^ However, such considerations are also critical for research, in ensuring the clear and accurate communication between genetic health specialists (of all kinds) and the public. The extent to which people can engage in informed discussion and deliberation, access services and express their needs in relation to these services is influenced not only by socioeconomic status, culture and religion but also by language.^67^ A recent competence framework published by Sharif et al. to enhance inclusion of diverse Asian communities in genomic research thus stresses the importance of cultural competence and awareness of language needs and preferences in enhancing engagement.^69^ Linguistic competence is important for the success of genomics not only in economically wealthy countries but also in developing countries, especially in an increasingly globalised world. People can only meaningfully engage with genomics when genomics and its concepts can be communicated to them (in research, genetic counselling, etc.), and they, in turn can communicate in a linguistically equivalent manner.

## Conclusions

Achieving genomic medicine, based on genomic research, that works for everyone requires two things. The first is an acknowledgement that a fair and just genomic medicine service can only be delivered if the data that guides genomic variant interpretation is populated by DNA from ancestrally diverse people. The global genomics community has made some progress in this direction, but far more needs to be done. The second is distribution, taking steps to ensure that the advantages that accrue from genomics do not only benefit those who are currently well represented in genomic datasets. Central to this is the need to engage public groups globally, to understand their concerns and hesitations related to genomic data collection, and to act in relation to these concerns to build genomic research and medicine initiatives that are worthy of public trust. In doing so, it is important to recognise that being trustworthy is a necessary step in establishing public trust in genomics research. It is not, however, ever likely to be sufficient to *ensure* public trust. Nevertheless, by working with communities to establish the qualities of trustworthy research, by making it clear to both public and researchers what these qualities are, and by striving to meet them, genomics research can aim to put itself in a position where it is seen to merit trust, and where misplaced trust – and the damage it causes to the research endeavour – can be minimised.

## Outstanding questions

Future research needs to focus on gathering the evidence needed to guide conversations between researchers and communities around power and privilege. Funding and resources must be put towards the social science and ethics research needed to understand how power dynamics affect participation in research and relations of trust, but also counselling and communication research to create practical tools that lead to meaningful and positive outcomes for all stakeholders.

## Contributors

JA did the literature search for this review and wrote the manuscript. RM, AC, CP and AM reviewed, edited and provided counsel on the manuscript. In addition, AM collated all revisions, proofed and submitted the final manuscript. All authors have read and approved the final version of the manuscript.

## Declaration of interests

The authors declare no conflicts of interests.

## References

[1] Stark Z., Dolman L., Manolio T.A. (2019). Integrating genomics into healthcare: a global responsibility. Am J Hum Genet.

[2] Popejoy A.B., Fullerton S.M. (2016). Genomics is failing on diversity. Nature.

[3] Bentley A.R., Callier S., Rotimi C.N. (2017). Diversity and inclusion in genomic research: why the uneven progress?. J Commun Genet.

[4] Zhang Y., Li Q., Wang X., Zhou X. (2015).

[5] Denny J.C., Rutter J.L., Goldstein D.B. (2019). The “all of us” research program. N Engl J Med.

[6] European Commission. DECLARATION OF COOPERATION: Towards access to at least 1 million sequenced genomes in the European Union by 2022. 2018. https://www.euapm.eu/pdf/EAPM_Declaration_Genome.pdf

[7] Gurdasani D., Carstensen T., Tekola-Ayele F. (2015). The African Genome Variation Project shapes medical genetics in Africa. Nature.

[8] Choudhury A., Aron S., Botigué L.R. (2020). High-depth African genomes inform human migration and health. Nature.

[9] Coles E., Mensah G.A. (2017). Geography of genetics and genomics research funding in Africa. Glob Heart.

[10] Tindana P., Yakubu A., Staunton C. (2019). Engaging research ethics committees to develop an ethics and governance framework for best practices in genomic research and biobanking in Africa: the H3Africa model. BMC Med Ethics.

[11] Tindana P., Depuur C., de Vries J., Seeley J., Parker M. (2020). Informed consent in genomic research and biobanking: taking feedback of findings seriously. Glob Bioeth.

[12] Wonkam A. (2021). Sequence three million genomes across Africa. Nature.

[13] Robertson S., Hindmarsh J., Berry S. (2018). Genomic medicine must reduce, not compound, health inequities: the case for Hauora- enhancing genomic resources for New Zealand. N Z Med J.

[14] Hudson M., Garrison N.A., Sterling R. (2020). Rights, interests and expectations: indigenous perspectives on unrestricted access to genomic data. Nat Rev Genet.

[15] Gibbs R.A., Boerwinkle E., Doddapaneni H. (2015). A global reference for human genetic variation. Nature.

[16] Mills M.C., Rahal C. (2020). The GWAS diversity monitor tracks diversity by disease in real time. Nat Genet.

[17] (2017). Comparison of sociodemographic and health-related characteristics of UK Biobank participants with those of the general population. Am J Epidemiol.

[18] Fry A., Littlejohns T.J., Sudlow C. (2017). Comparison of sociodemographic and health-related characteristics of UK Biobank participants with those of the general population. Am J Epidemiol.

[19] Kim M.S., Patel K.P., Teng A.K., Berens A.J., Lachance J. (2018). Genetic disease risks can be misestimated across global populations. Genome Biol.

[20] Duncan L., Shen H., Gelaye B. (2019). Analysis of polygenic risk score usage and performance in diverse human populations. Nat Commun.

[21] United Nations (1948).

[22] Office for Human Research Protections (2016). https://www.hhs.gov/ohrp/regulations-and-policy/belmont-report/index.html.

[23] Reardon J. (2020). Why and how bioethics must turn toward justice: a modest proposal. Hastings Cent Rep.

[24] Nielsen R., Akey J.M., Jakobsson M., Pritchard J.K., Tishkoff S., Willerslev E. (2017). Tracing the peopling of the world through genomics. Nature.

[25] Ramani P.K., Sankaran B.P. (2021). Tay-Sachs disease. J Neonatal Nurs.

[26] Scotet V., L'Hostis C., Férec C. (2020). The changing epidemiology of cystic fibrosis: incidence, survival and impact of the CFTR gene discovery. Genes.

[27] Angelucci E., Burrows N., Losi S., Bartiromo C., Hu X.H. (2016). Beta-Thalassemia (BT) prevalence and treatment patterns in Italy: a survey of treating physicians. Blood.

[28] Freedman B.I., Limou S., Ma L., Kopp J.B. (2018). APOL1-associated nephropathy: a key contributor to racial disparities in CKD. Am J Kidney Dis.

[29] Cuypers B., Lecordier L., Meehan C.J. (2016). Apolipoprotein L1 variant associated with increased susceptibility to trypanosome infection. mBio.

[30] Ku E., Lipkowitz M.S., Appel L.J. (2017). Strict blood pressure control associates with decreased mortality risk by APOL1 genotype. Kidney Int.

[31] Narang P., Johnson A., Enja M., Lippmann S. (2016). Pharmacogenomics can enhance prescribing of psychiatric medications. South Med J.

[32] Abraham S., Jethwa H. (2017). Pharmacogenomics: the scientific basis of rational drug development and prescribing. Clin Pharmacol Biopharm.

[33] Guedeney P., Giustino G., Sorrentino S. (2019). Efficacy and safety of alirocumab and evolocumab: a systematic review and meta- analysis of randomized controlled trials. Eur Heart J.

[34] Cohen J.C., Boerwinkle E., Mosley T.H., Hobbs H.H. (2006). Sequence variations in PCSK9, low LDL, and protection against coronary heart disease. N Engl J Med.

[35] Wood L.D., Parsons D.W., Jones S. (2007). The genomic landscapes of human breast and colorectal cancers. Science.

[36] Ray K.K., Wright R.S., Kallend D. (2020). ORION-10 and ORION-11 investigators. Two phase 3 trials of inclisiran in patients with elevated LDL cholesterol. N Engl J Med.

[37] Sirugo G., Williams S.M., Tishkoff S.A. (2019). The missing diversity in human genetic studies. Cell.

[38] Manrai A.K., Funke B.H., Rehm H.L. (2016). Genetic misdiagnoses and the potential for health disparities. N Engl J Med.

[39] Khera A.V., Chaffin M., Aragam K.G. (2018). Genome-wide polygenic scores for common diseases identify individuals with risk equivalent to monogenic mutations. Nat Genet.

[40] Martin A.R., Kanai M., Kamatani Y., Okada Y., Neale B.M., Daly M.J. (2019). Clinical use of current polygenic risk scores may exacerbate health disparities. Nat Genet.

[41] (2018). Polygenic risk score for schizophrenia is more strongly associated with ancestry than with schizophrenia. Psychiatr Genet.

[42] Cornel M.C., Bonham V.L. (2017). Genomics for all in the 21st century?. J Commun Genet.

[43] Pearce C., Goettke E., Hallowell N., McCormack P., Flinter F., McKevitt C. (2019). Delivering genomic medicine in the United Kingdom National Health Service: a systematic review and narrative synthesis. Genet Med.

[44] Middleton A., Niemiec E., Prainsack B. (2018). Your DNA, Your Say”: global survey gathering attitudes toward genomics: design, delivery and methods. Per Med.

[45] Middleton A., Milne R., Almarri M.A. (2020). Global public Perceptions of genomic data sharing: what shapes the willingness to donate DNA and health data?. Am J Hum Genet.

[46] Milne R., Morley K.I., Howard H. (2019). Trust in genomic data sharing among members of the general public in the UK, USA, Canada and Australia. Hum Genet.

[47] Dive L., Critchley C., Otlowski M. (2020). Public trust and global Biobank networks. BMC Med Ethics.

[48] Scherr C.L., Ramesh S., Marshall-Fricker C., Perera M.A. (2019). A review of African Americans’ beliefs and attitudes about genomic studies: opportunities for message design. Front Genet.

[49] Mauffrey C., Giannoudis P., Civil I. (2017). Pearls and pitfalls of open access: the immortal life of Henrietta Lacks. Injury.

[50] Mello M.M., Wolf L.E. (2010). The Havasupai tribe case — lessons for research involving stored biologic samples. N Engl J Med.

[51] Lee S.S.J., Cho M.K., Kraft S.A. (2019). “I don't want to be Henrietta lacks”: diverse patient perspectives on donating biospecimens for precision medicine research. Genet Med.

[52] Yeh V.M., Bergner E.M., Bruce M.A. (2020). Can precision medicine actually help people like me? African American and hispanic perspectives on the benefits and barriers of precision medicine. Ethn Dis.

[53] Moodley K., Sibanda N., February K., Rossouw T. (2014). “It's my blood”: ethical complexities in the use, storage and export of biological samples: perspectives from South African research participants. BMC Med Ethics.

[54] Smith D. Pfizer pays out to Nigerian families of meningitis drug trial victims | Nigeria | The Guardian. Guard 2011. https://www.theguardian.com/world/2011/aug/11/pfizer-nigeria-meningitis-drug-compensation Accessed 10 September 2021.

[55] Fullwiley D., Gibbon S., Gibbon S., Prainsack B., Hilgartner S., Lamoreaux J. (2018). Routledge Handbook of Genomics, Health and Society.

[56] Turnbull D. (2000).

[57] Haas F. (2008). German science and black racism—roots of the Nazi Holocaust. FASEB J.

[58] de Vries J., Pepper M. (2012). Genomic sovereignty and the African promise: mining the African genome for the benefit of Africa. J Med Ethics.

[59] Haelewaters D., Hofmann T.A., Romero-Olivares A.L. (2021). Ten simple rules for Global North researchers to stop perpetuating helicopter research in the Global South. PLOS Comput Biol.

[60] Serwadda D., Ndebele P., Grabowski M.K., Bajunirwe F., Wanyenze RK. (2018). Open data sharing and the Global South—who benefits?. Science.

[61] Wonkam A.M.A., Kenfack M., Muna W., Ouwe-Missi-Oukem-Boyer O. (2011). Ethics of human genetic studies in Sub-Saharan Africa: the case of Cameroon through a bibliometric analysis. Dev World Bioeth.

[62] Milne R., Morley K.I., Almarri M.A. (2021). Demonstrating trustworthiness when collecting and sharing genomic data: public views across 22 countries. Genome Med.

[63] Ginther D.K., Schaffer W.T., Schnell J. (2011). Race, ethnicity, and NIH research awards. Science.

[64] (2016). Science and inequality. Nature.

[65] Hoppe T.A., Litovitz A., Willis K.A. (2019). Topic choice contributes to the lower rate of NIH awards to African-American/black scientists. Sci Adv.

[66] Nooruddin M., Scherr C., Friedman P. (2020). Why African Americans say “no”: a study of pharmacogenomic research participation. Ethn Dis.

[67] Jao I., Kombe F., Mwalukore S. (2015). Research stakeholders’ views on benefits and challenges for public health research data sharing in Kenya: the importance of trust and social relations. PLoS One.

[68] Middleton A., Emery S.D., Turner G.H. (2010). Views, knowledge, and beliefs about genetics and genetic counseling among deaf people. Sign Lang Stud.

[69] Sharif S.M., Blyth M., Ahmed M. (2020). Enhancing inclusion of diverse populations in genomics: a competence framework. J Genet Couns.

